# Expression of Neural Crest Markers GLDC and ERRFI1 is Correlated with Melanoma Prognosis

**DOI:** 10.3390/cancers11010076

**Published:** 2019-01-11

**Authors:** Katharina Jäger, Lionel Larribère, Huizi Wu, Christel Weiss, Christoffer Gebhardt, Jochen Utikal

**Affiliations:** 1 Skin Cancer Unit, German Cancer Research Center (DKFZ), D-69121 Heidelberg, Germany; rina.jaeger@yahoo.de (K.J.); huizijiaguo125@gmail.com (H.W.); Ch.gebhardt@uke.de (C.G.); j.utikal@dkfz.de (J.U.); 2 Department of Dermatology, Venereology and Allergology, University Medical Center Mannheim, Ruprecht-Karl University of Heidelberg, 68167 Mannheim, Germany; 3 Department of Clinical Pharmacology, Central South University, Changsha 410083, China; 4 Institute for Medical Statistics, Medical Faculty Mannheim, Ruprecht-Karl University of Heidelberg, 68167 Mannheim, Germany; Christel.weiss@medma.uni-heidelberg.de; 5 Department of Dermatology and Venereology, University Hospital Hamburg-Eppendorf (UKE), 20246 Hamburg, Germany

**Keywords:** melanoma, neural crest cells, marker, prognosis

## Abstract

Regulation of particular genes during the formation of neural crest (NC) cells is also described during progression of malignant melanoma. In this context, it is of paramount importance to develop neural crest models allowing the identification of candidate genes, which could be used as biomarkers for melanoma prognosis. Here, we used a human induced Pluripotent Stem Cells (iPSC)-based approach to present novel NC-associated genes, expression of which was upregulated in melanoma. A list of 8 candidate genes, based on highest upregulation, was tested for prognostic value in a tissue microarray analysis containing samples from advanced melanoma (good versus bad prognosis) as well as from high-risk primary melanomas (early metastasizing versus non or late-metastasizing). CD271, GLDC, and ERRFI1 showed significantly higher expression in metastatic patients who died early than the ones who survived at least 30 months. In addition, GLDC and TWIST showed a significantly higher immunohistochemistry (IHC) score in primary melanomas from patients who developed metastases within 12 months versus those who did not develop metastases in 30 months. In conclusion, our iPSC-based study reveals a significant association of NC marker GLDC protein expression with melanoma prognosis.

## 1. Introduction

Malignant melanoma is a malignant tumor originating from the melanocytic cell system. Due to the tendency for metastasis, melanoma is a very aggressive skin cancer showing a high mortality index in advanced stages [1]. Melanocytes are pigment-producing cells of the skin originating from neural crest cells (NC cells). During embryonic development, pluripotent stem cells differentiate into multipotent NC cells, which can further differentiate into melanoblasts and finally mature into melanocytes [2]. Recent studies suggest a regain of NC properties in malignant melanoma cells, which are able to sustain the tumor growth and promote metastasis [3,4,5]. It is therefore important to get a better understanding of the NC-associated genes and their associated function in melanoma pathogenesis.

Tumor thickness, ulceration, and metastasis in regional lymph nodes are major risk factors in melanoma patients and are used as prognostic markers [6]. Unfortunately, these markers often do not predict accurately the patient’s outcome. In order to predict the prognosis more precisely, a better understanding of melanoma genesis and its marker and mechanisms is needed. By using gene expression profiling approaches, we first display in this current article a number of genes which are not only expressed in NC cells but also overly expressed in melanoma and investigated their ability to state a prognosis for clinical daily use. Additionally, CD271 and Ki67 were investigated regarding the patients’ prognosis. CD271 is a known NC cell marker, which seems to play an important role in invasion and migration processes of melanoma [3]. As a known proliferation marker, Ki67 can be used as a predictive and prognostic marker in tumor tissue [7].

## 2. Results

### 2.1. Detection of Novel Neural Crest Cell-Associated Markers in Melanoma Cells

Many investigations have now shown a reactivation of the embryonic neural crest (NC) signaling pathways during melanomagenesis and during tumor progression to metastasis. The tumor heterogeneity could be described by the presence in the tumor of different clonal subpopulations of cells characteristic of which is to present an undifferentiated and highly invasive phenotype [8]. For example, NC-related gene TWIST1 is highly expressed in metastatic melanoma [9] and expression of MSX1 in melanocytes induced a dedifferentiated phenotype [10].

Our aim was therefore to identify new candidate genes, expression of which was upregulated both in NC cells and in melanoma cells (compared to normal melanocytes). For this purpose, we were able to differentiate human induced Pluripotent Stem Cells (hiPSCs) into NC cells according to a protocol we have set up in the laboratory [11]. The transcriptome profile of these NC cells was compared to that of parental hiPSCs and top upregulated genes were selected. We also compared the transcriptome profile of melanoma cell lines to normal human melanocytes (NHM) and selected top regulated genes.

**Figure 1:**
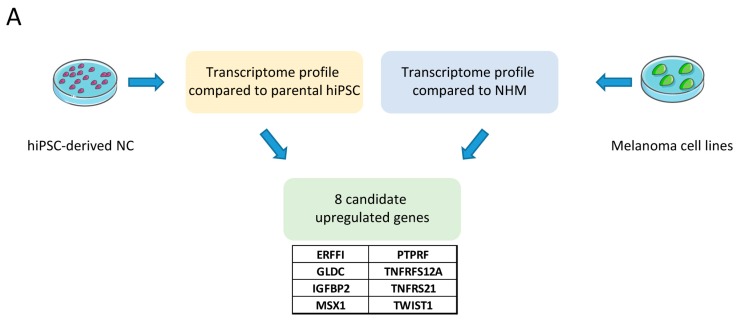

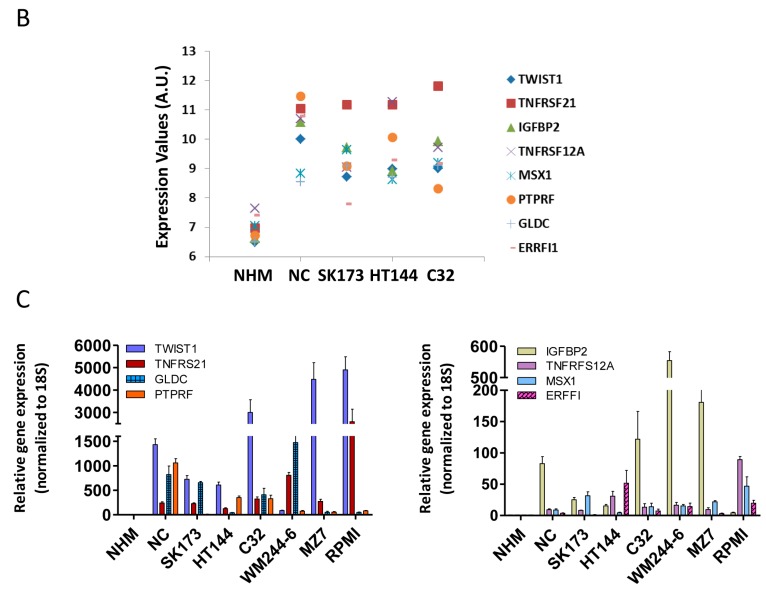
Detection of novel neural crest cell associated markers in melanoma cells. (**A**) Schematic of the workflow. Transcriptome of neural crest (NC) cells and melanoma cell lines were compared to either parental human induced Pluripotent Stem Cells (hiPSCs) or normal human melanocytes (NHM). The 8 most upregulated genes in both conditions were selected. (**B**) Gene expression values of the 8 candidate markers from the transcriptome analysis in NC and melanoma cell lines compared to NHM. (**C**) qPCR-based gene expression of the 8 candidate markers in NC and melanoma cell lines compared to NHM. rRNA 18S was used as an endogenous expression control. Data are shown as mean ± SD of biological triplicates.

We focused on a list of 8 candidate genes including two genes known to be expressed in NC (TWIST1 and MSX1) and six new genes (TNFRSF12A, GLDC, ERRFI, IGFBP2, PTPRF) (Figure 1A). Compared to the average expression value of 3 independent NHM samples, the expression values of all candidate genes from the transcriptome analysis were significantly upregulated in NC and in melanoma cells (log2-fold change > 1) (Figure 1B). As a confirmation of the microarray data, we analyzed the candidate gene’s expression by qPCR and observed a significant increase in six melanoma cell lines as well as in the NC cells when compared to NHM (Figure 1C). Because of the high variation in expression between cell lines, some genes showed high upregulation (TWIST1, TNFRSF21, GLDC, and PTPRF, left panel) and some others showed lower upregulation (IGFBP2, TNFRSF12A, MSX1, and ERFFI, right panel).

### 2.2. High CD271, GLDC, and ERRFI1 Protein Expression is Associated with Reduced Survival in Stage IV Melanoma Patients

**Figure 2:**
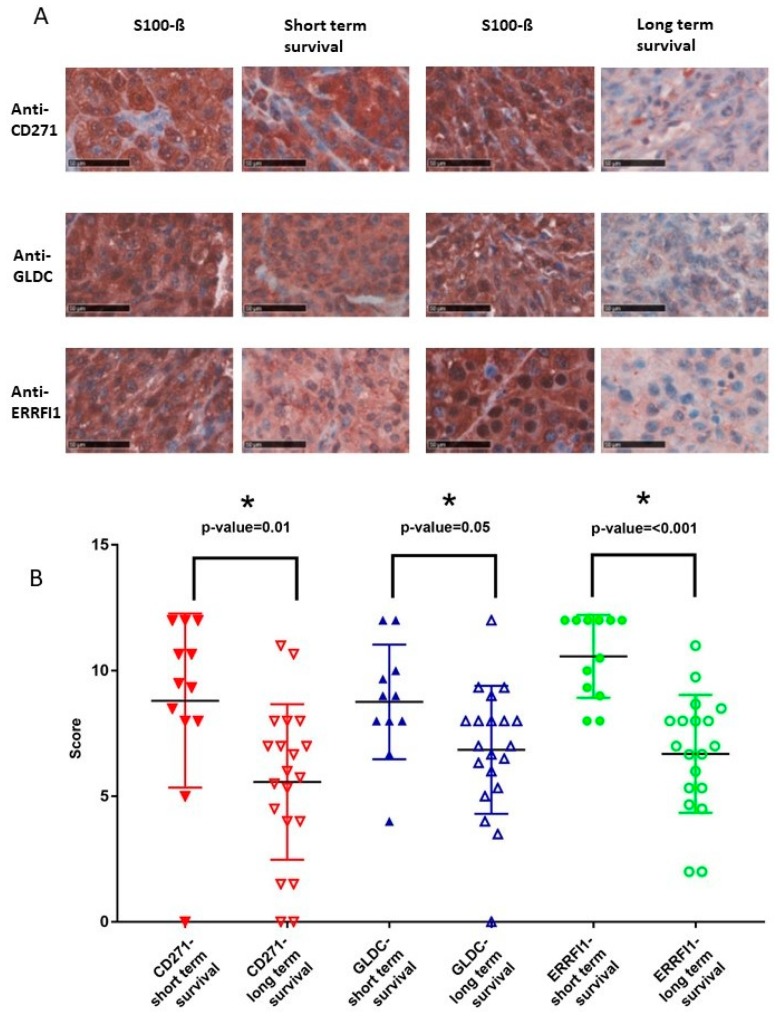
Neural crest marker CD271, GLDC, and ERRFI1 are highly expressed in advanced melanoma patients with bad prognosis. (**A**) Immunohistochemical stainings for CD271, GLDC, and ERRFI1 for short-term survival and long-term survival. Scale bar: 50 µm. (**B**) Overall IHC score analyses for CD271, GLDC, and ERRFI1 for short- and long-term survival. Y-axis shows the overall IHC score (0–12), which was calculated from the means of the products of quantity and intensity of the stainings. The displayed antibodies showed *p*-values < 0.05 (*) analyzed with two-tailed *t*-test.

Tumor samples from patients who died within 12 months (survival group A) were compared to tumor samples from patients who survived at least 30 months (survival group B) after distant metastasis. Samples from survival group A showed a significantly higher mean overall immunohistochemistry (IHC) score for Anti-CD271, Anti-GLDC, and Anti-ERRFI1 (*p*-value < 0.05) (Figure 2). In survival group A, the mean overall IHC for Anti-CD271 was 8.81 compared to 5.57 in survival group B, *p*-value = 0.01. Anti-GLDC showed an overall IHC score of 8.76 for survival group A vs. 6.85 in survival group B, *p*-value = 0.05. For the antibody against ERRFI1, the mean overall IHC score was 10.57 in survival group A and 6.69 in survival group B, *p*-value < 0.001.

Samples from survival group A showed a higher mean overall IHC score for Anti-MSX1, Anti-TWIST, Anti-Ki67, Anti-PTPRF, Anti-TNFRSF21, and Anti-TNFRSF12a but without significance (Figure S1). Anti-MSX1 showed in survival group A a mean overall IHC score of 9.83 and 7.79 in survival group B, *p*-value = 0.07. In survival group A, the mean overall IHC score for Anti-TWIST was 9.41 and 8.31 in survival group B, *p*-value = 0.29. The mean overall IHC score of Ki67 was 10.14 in survival group A and 8.58 in survival group B. In survival group A, the mean overall IHC score of Anti-PTPRF was 7.49 vs. 6.19 in survival group B, *p*-value = 0.28. Anti-TNFRSF21 showed a mean overall IHC score of 9.03 in survival group A and 8 in survival group B, *p*-value = 0.41. In survival group A, the mean overall IHC score of Anti-TNFRSF12a was 9.31 versus 8.07, *p*-value = 0.3. Anti-IGFBP2 showed in survival group A a mean overall IHC score of 5.07 versus 5.19 in survival group B, *p*-value = 0.91 (Table 1 and Table 2).

According to our study, antibodies against CD271, GLDC, and ERRFI1 could be used as a tool to predict prognosis of overall survival at stage IV disease of malignant melanoma. Malignant melanoma patients with tumor samples with an IHC score > 8 for Anti-CD271, >8 for Anti-GLDC, and >9 for Anti-ERRFI1 are more likely to die early and therefore show a poorer prognosis after diagnosis stage IV in comparison with patients with smaller IHC scores for the described antibodies.

### 2.3. High TWIST and GLDC Protein Expression are Associated with Metastasis Development in High-Risk Primary Melanomas

**Figure 3:**
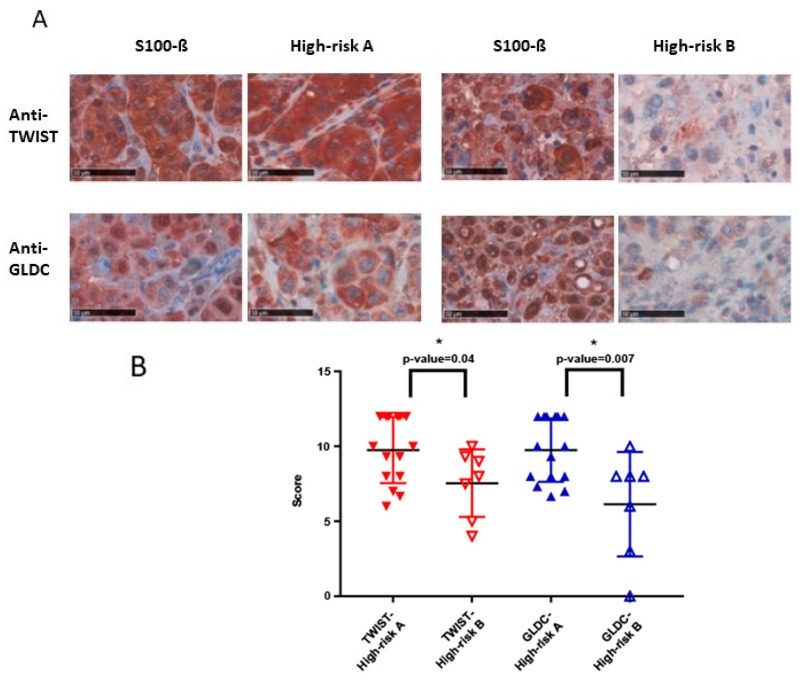
TWIST and GLDC high protein expressions are associated with metastasis development in high-risk primary melanomas. (**A**) Immunohistochemical stainings of high-risk primary melanomas are shown for TWIST and GLDC in high-risk group A and high-risk group B. Scale bar: 50 µm (**B**) Overall IHC score analyses of the two subgroups (high-risk group A and high-risk group B) are displayed in this chart. Y-axis shows the overall IHC score (0–12), which was calculated from the means of the products of quantity and intensity of the stainings. The displayed antibodies showed *p*-values < 0.05 (*) analyzed with two-tailed *t*-test.

Tumor samples of primary melanoma from patients who were at high risk to metastasize (primary lesion ≥ 2 mm) were compared regarding the time until metastasis occurred. In high-risk group A, patients were included who metastasized in 12 months. Patients who did not metastasize in 30 months or longer were included in the second group (high-risk group B). Due to low sample number (*n* = 7), we assumed a non-normal distribution in high-risk group B. Samples from high-risk group A showed a significantly higher mean overall IHC score for Anti-TWIST and Anti-GLDC in comparison to high-risk group B (Figure 3). The mean overall IHC score for anti-TWIST was 9.76 in high-risk group A, compared to 7.55 in high-risk group B, *p*-value = 0.04. Anti-GLDC showed a mean overall IHC score of 9.76 in high-risk group A versus 6.14 in high-risk group B, *p*-value = 0.007.

Tumor samples from high-risk group A showed a higher mean overall IHC score for Anti-MSX1, Anti-CD271, Anti-ERRFI1, Anti-PTPRF, Anti-TNFRSF21, Anti-TNFRSF12a, and Anti-IGFBP2, but without significance (Figure S2). The mean overall IHC score for Anti-MSX1 was 9.56 in high-risk group A and 8.75.

In high-risk group B, *p*-value = 0.5. Anti-CD271 showed a mean overall IHC score of 8.79 in high-risk group A vs. 7.67 in high-risk group B, *p*-value = 0.37. The mean overall IHC score in high-risk group A was 9.23 and 7.28 in high-risk group B for Anti-ERRFI1, *p*-value = 0.35. For Anti-Ki67, the mean in high-risk group A was 7.47 versus 8.80 in high-risk group B. Anti-PTPRF showed a mean overall IHC score of 7.71 in high-risk group A vs. 7.12 in high-risk group B, *p*-value = 0.67, and 9.89 was the mean overall IHC score in high-risk group A and 8.63 in high-risk group B for Anti-TNFRSF21, *p*-value = 0.21. For Anti-TNFRSF12a, the mean overall IHC score was 10.33 in high-risk group A versus 9.38 in high-risk group B, *p*-value = 0.25. In high-risk group A, the mean overall IHC score was 13.53 versus 8.5 for Anti-IGFBP2 (Table 3 and Table 4).

## 3. Discussion

Next to tumor thickness and ulceration of primary melanoma, the extent of regional and distant metastasis only lactate dehydrogenase (LDH) is used as a prognostic marker in the TMN classification system of the American Joint Committee on Cancer (AJCC) 2017. However, these markers are used for staging and often do not predict accurately the patient’s outcome. LDH is only used as a prognostic marker in clinic when distant metastasis already occurred [12]. Additionally, LDH is not specific for malignancy as it is also elevated in cases of cell damage, inflammatory processes, and hemolysis [13]. In our study, we show that genes that are highly expressed not only in neural crest cells but also in malignant melanoma cells are associated with prognosis. Upregulation of CD271, ERRFI1, and GLDC is in line with a bad prognosis. Additionally, upregulation of TWIST and GLDC is associated with early metastasis and therefore can be used as prognostic markers before metastasis occurred.

The role of CD271 in tumor transformation and progression has been widely investigated. It has also been suggested as a marker of cancer stem cell-like population in human melanoma tissues [3]. The results of these studies are, however, not always on the same line. CD271 has been described as a dual mediator by suppressing melanoma cell proliferation and promoting metastasis while highly expressed in malignant melanoma cells [14]. Furthermore, CD271 was identified as a T cell-suppressive molecule and, therefore, might be interesting for antimelanoma immunotherapy [15]. However, a study based on xenograft transplantations derived from 7 melanoma patients showed variable expression level of CD271 after propagation of the same tumor into different mice. In addition, no correlation was found between the expression of CD271 and the tumorigenicity of the samples [16,17]. Our results show that a higher expression of CD271 is associated with a poor prognosis. The cut-off point for Anti-CD271 was 8 overall IHC score. For patients with an overall IHC score > 8 there is a 12 times greater risk to belong in survival group A and, therefore, to die earlier. The validity of this model is 80%. Because this marker is not commonly accepted, these data have to be taken with caution.

As part of a multienzyme complex, glycine decarboxylase (GLDC) is involved in the biosynthesis of serine. It has been shown that GLDC is a metabolic oncogene and its expression is strongly correlated with rates of cancer proliferation and greater mortality, e.g., in breast cancer [18,19]. Our study reveals that a greater expression, which can be seen in a greater overall IHC score, is in line with a poor prognosis. The calculated cut-off point for Anti-GLDC was also 8. Patients with an overall IHC score > 8 are 4.5 times more likely to be part of survival group A. The validity of this model is 73.6%. In addition, our results show that a greater expression of the GLDC protein (IHC score > 9.33) in primary melanoma increases the risk for early metastasis. Patients with an IHC score > 9.33 are 5.7 times more likely to belong to high-risk group A and, therefore, develop a metastasis within a shorter term. The validity of this model is 96%.

ERRFI1 is known as a tumor suppressor by directly inhibiting the epidermal-growth-factor-receptor and, therefore, its downstream pathways initializing cell growth [20,21]. In addition, ERFFI1 inhibits another receptor family (Erbb), activation of which leads to cell survival, proliferation, migration, and invasion [22]. It has been shown that ERRFI1 alters negatively invasion and migration processes in malignant melanoma. However, new findings suggest that the role of ERRFI1 depends on the EGFR level. Therefore, the downregulation of ERRFI1 in an EGFR low environment leads to a higher migration rate and promotes cellular growth [23,24]. In our work, the EGFR level was not determined, so further investigations are needed to evaluate the validity of ERRFI1 as a prognostic marker in different subtypes of malignant melanoma. The calculated cut-off point for the antibody against ERRFI1 was 9. Patients with an overall IHC score > 9 are 17 times more likely to belong in survival group A. The validity of the model is 92.1%.

MSX1 is a transcription factor, which plays a part in the epithelial-to-mesenchymal transition during the embryonic development of the melanocyte lineage from the neural crest cells [25]. It could be shown that reactivation of the transcription factor MSX1 in melanoma cells leads to neural crest precursor-like properties, like migration and plasticity [10]. In our studies, no significant difference could be shown, but patients with a poorer prognosis did show a higher IHC (*p*-value = 0.07). A bigger population group would be needed in order to clarify if MSX1 is usable as a significant tool. TWIST, like MSX1, is a transcription factor, which is part of the epithelial-to-mesenchymal transition. It therefore plays an important role in cell movement, invasion, and survival and is often overexpressed in cancer diseases [26]. It has been shown that TWIST especially promotes metastasis processes and has no influence of primary melanoma growth [27,28,29]. Similar to MSX1, TWIST is involved in metastasis processes. In our study, primary melanomas which are at great risk to metastasis (tumor thickness ≥ 2 mm) with a greater TWIST expression showed earlier metastasis compared to those with lower expression. The calculated cut-off point for TWIST was IHC > 12, the validity for this model is 75.2%. As 12 is the maximal IHC score that can be reached, further investigations are needed to specify the cut-off point as a clinical tool. In addition, there has been a difference for Anti-TWIST between survival group A and survival group B, but without significance. Further investigations are needed in order to display an increasing development of MSX1 and TWIST expression during tumor progression.

Ki67 is not to be found in neural crest cells but is an established proliferation marker, which is expressed in dividing cells [30]. It is used as a predictive and prognostic marker, e.g., in breast cancer [7]. As described above, there is a non-significant difference in Ki67 expression between the two groups.

PTPRF encodes the tyrosine-phosphate-receptor-type F, which controls EGFR signaling. PTPRF is overexpressed in primary melanoma and metastasis of malignant melanoma [31]. The fact that PTPRF is not only overexpressed in metastasis but also in primary melanoma suggests that there is no relevant trend in expression during tumor progression. In our studies, no significant difference in PTPRF expression between the two groups was shown.

TNFRSF21 and TNFRSF12a are members of the tumor necrosis receptor family and contribute to the activation of NF-kappaB, which plays a role in tumorigenesis [32,33]. The IHC scores of the two groups for both antibodies suggest a greater expression during tumor progression; therefore, further investigations are needed in order to confirm the validity.

Finally, IGFBP2 is a tool for proliferation, apoptosis, and migration of cells during embryonic development and tumor cells [34,35]. However, Anti-IGFBP2 did not show a significant difference between the two groups investigated in our study.

One limitation of this study is the small-analyzed population size. In order to improve validity of this study, a bigger population group would be needed. Also, gender distribution, especially in the high-risk group, does not fit the normal distribution in Germany [36]. Here might be a bias as male patients have a shorter overall survival and a greater risk for metastasis compared to women [37,38]. In high-risk group A, patients were included which were already metastasized at time of diagnosis. In these cases, it cannot be certainly said how many months have passed between occurrence of tumor and metastasis. However, considering experience with course of disease and tumor parameter thickness, the time in-between is negligible in these cases.

## 4. Materials and Methods

### 4.1. Cell Lines

Human melanoma cell lines (C32, HT144, SKmel173, WM266-4, MZ7, and RPMI) were cultured in DMEM (Gibco, Life Technologies, Darmstadt, Germany) with 10% FBS (Biochrom, Berlin, Germany), 0.1 mM β-mercapthoethanol (Gibco, Life Technologies), 1% non-essential amino acids (NEAA) and 1% Penicillin/Streptomycin (Sigma-Aldrich, St. Louis, MO, USA). Normal human melanocytes (NHM) were isolated from donor foreskins according to the ethical regulation (Ethics committee II, University Medical Center Manheim, Mannheim, Germany) and were cultivated in medium 254 (Gibco, Life Technologies) supplemented with 100× human melanocyte growth supplement (HMGS) (Gibco, Life Technologies). Human neural crest cells were derived from hiPSC and cultured in the same medium as for melanoma cell lines.

### 4.2. RNA Isolation and cDNA Synthesis

Total RNA isolation from all melanoma cell lines (C32, HT144, SKmel173, WM266-4, MZ7, and RPMI), human neural crest cells (NC) and normal human melanocytes (NHM) was done using RNeasy Mini kit (Qiagen, Düsseldorf, Germany) according to the manufacturer’s protocol. The RNA was treated with DNase I on the column. RNA concentration and quality were measured by NanoDrop ND1000 spectrophotometer. cDNA was synthesized using the Revert Aid First Strand cDNA synthesis kit (Thermo scientific, Darmstadt, Germany) according to the manufacturer’s protocol.

### 4.3. Microarray Gene Expression Profiling

Biotinylated cRNA was hybridized to whole-genome BeadChip Sentrix arrays HumanHT-12 v4 from ILLUMINA (Santa Clara, CA, USA) following the manufacturer’s indications. Microarray scanning was carried out using an iScan array scanner. As test for significance, a Bayes test was used on the bead expression values of the two groups of interest. The average expression value is calculated as mean of the measured expressions of beads together with the standard deviation of the beads. After selecting the genes, which *p*-values were inferior to 0.05, log2-expression values of the differentially expressed genes were represented.

Gene expression datasets were uploaded on GEO database: GSE123686.

### 4.4. qPCR

Quantitative real-time PCR (qPCR) was performed using SYBR Green (Applied Biosystems, Life technologies, Darmstadt, Germany) on a 7500 real-time PCR system (Applied Biosystems, Life technologies). In all experiments, rRNA 18s was used as the housekeeping gene and the values were normalized to it. Relative gene expressions were quantified by calculating (∆∆Ct). Primers used are as follow (Table 5):

### 4.5. Patient’s Sample

In this study, histopathologic tumor samples (primary melanoma and metastasis) of patients diagnosed with malignant melanoma between 1989 and 2013 were collected and assigned into two groups. All patients did not receive novel immunotherapeutic or targeted therapeutic drugs that have an impact on overall survival. This study was performed in accordance to the ethical vote (2014-835R-MA).

In the first group (survival group A) were included tumor samples of patients who died within 12 months after diagnosis of stage IV melanoma (*n* = 12). Only metastases were analyzed. Patients who survived 30 months or longer were included in the second group (*n* = 20; survival group B). In survival group A, eight patients were male and four female. The mean age at initial diagnosis in the first group was 52 years. The mean Breslow tumor thickness of primary melanoma in Survival group A was 2.8 mm. In survival group B, the mean age at initial diagnosis was 61 years. Nine patients were male and eleven female. The mean Breslow tumor thickness of primary melanoma in survival group B was 3.3 mm (see also Table 6). Classifications were made according to system of the American Joint Committee on Cancer (AJCC) of 2009.

In addition, tumor samples of patients with tumors ≥ 2 mm showing high risks to metastasize were identified and assigned to two groups. In the first group (high-risk group A), patients were included who developed metastasis within the first 12 month (*n* = 16) and the second group contained patients who did not metastasize within 30 months (*n* = 7; high-risk group B). In this group, only primary melanomas were analyzed. In high-risk group A, four patients were female and twelve patients male. The mean age in this group at initial diagnosis was 76 years. The mean Breslow tumor thickness of primary melanoma in this group was 5 mm. In high-risk group B, five patients were female and two male. The mean age at initial diagnosis was 68 years and the mean Breslow tumor thickness was 2.5 mm (see also Table 7).

### 4.6. Tissue Microarray

Representative tumor areas were detected on HE sections and tissue punch samples (diameter 2 mm) were taken from paraffin-embedded tumor block and displayed on Tissue Microarrays (TMA) according to a previous report [39]. For one tumor sample up to four punches were taken.

### 4.7. Immunohistochemistry

An amount of 0.9 µm slices of the in paraffin-embedded tumors on the TMA were stained using standard protocols 11 times with the following antibodies: Anti-GLDC (Atlas Antibodies, Bromma, Sweden, HPA002318), Anti-CD271 (BP Pharmingen, Heidelberg, Germany, 557194), Anti-ERRIF1 (Atlas Antibodies HPA027206), Anti-MSX1 (abcam, Cambridge, UK, ab49153), Anti-TNFRSFR12A (Atlas Antibodies HPA007853), Anti-Ki67 (abcam ab1667), Anti-PTPRF (Atlas Antibodies HPA012710), Anti-TNFRSFR21 (Atlas Antibodies HPA006746), Anti-TWIST (abcam ab50581), Anti-IGFBP2 (Cell signaling, Denver, MA, USA, #3922), and Anti-S100 (Dako, Santa Clara, CA, USA, Z0311). As a negative control, stainings were made according to standard protocol without using primary antibodies. As secondary antibodies, the Dako EnVision™ System-HRP (Dako Kit, Rabbit K4009) was used for Anti-GLDC, Anti-ERRFI1, Anti-MSX1, Anti-TNFRSFR12a, Anti-Ki67, Anti-PTPRF, Anti-TNFRSFR21, Anti-TWIST, Anti-IGFBP2, and Anti-S100. The Dako EnVision™ System-HRP (Dako Kit, Mouse K4005) was used for Anti-CD271. For antibody dilution, Dako Antibody Diluents (Dako S0809) has been used. Antibody dilutions were made according to the manufacturers’ information sheet.

### 4.8. Analysis of Stained Tumor Samples and Statistics

Stained tumor samples were analyzed and an IHC (immunohistochemistry) score assigned to each sample.

IHC score was defined as the product of the immunopositivity score (0: <10%, 1: 10–25%; 2: 26–50%; 3: 51–75% and 4: >75%) and the immunointensity score (0: negative, 1: mild, 2: moderate, 3: strong). This score was made for each punch (min: 0; max: 12), except for antibody Anti-Ki67. As described above, up to four punches of one tumor sample were taken. Out of IHC scores from each punch, a mean IHC score of each tumor sample was calculated. For an overall IHC score, the mean value form scoring of the two investigators was calculated. For samples stained with Anti-Ki67, the percentage of stained cell nuclei was determined. Two investigators performed scoring independently. One investigation was performed blinded to detect potential bias. Figure 2a and Figures S3 and S4 give examples for immunointensity scores for each antibody.

Overall IHC scores were calculated for all samples and data comparison from survival group A and survival group B, also from high-risk group A and high-risk group B were statistically analyzed with two-tailed *t*-test. As mean and median did not differ much, we could assume data normally distributed. For Anti-Ki67 and Anti-IGFBP in high-risk group A and B, analyses were made with Mann–Whitney *U*-Test, as in these cases the data was not normally distributed. Equality of variances was tested using *f*-test. For each significant antibody, a cut-off point was determined by using logistic regression in order to objectify the informative value of the IHC scores. It was shown that patients with an overall IHC above the cut-off point have a greater risk to belong to group A. Therefore, the risk was calculated using odds ratios. In order to validate this prediction, the association of predicted probabilities and observed responses was put in relation.

## 5. Conclusions

In conclusion, this study shows that genes highly expressed in both neural crest cells and malignant melanoma cells are associated with prognosis. Indeed, an upregulation of CD271, GLDC, and ERRFI1 in metastatic melanoma is in line with a bad prognosis. In addition, GLDC upregulation in primary melanoma correlates with a risk for early metastasis. Overall, these data highlight the benefit provided by hiPSC-based neural crest models to identify new prognosis markers in neural crest-derived tumors such as melanoma.

## Figures and Tables

**Table 1:** Immunohistochemistry (IHC) scores for each antibody (survival group).

Patient Number	Anti-CD271	Anti-GLDC	Anti-ERRFI1	Anti-TWIST	Anti-MSX1	Anti-Ki67	Anti-PTPRF	Anti-TNFRSF21	Anti-TNFRSF12A	Anti-IGFBP2
**Short-Term Survivors**										
1	8.50	8.00	9.00	8.75	8.50	3.00	7.00	9.00	11.00	2.67
2	9.50	9.00	10.00	9.00	10.00	8.75	3.75	8.00	10.00	1.50
3	12.00	12.00	10.50	12.00	10.50	3.00	8.00	12.00	12.00	4.00
4	12.00	8.00	12.00	12.00	12.00	2.75	10.00	11.00	9.75	3.00
5	12.00	9.00	12.00	9.00	12.00	1.00	12.00	12.00	12.00	8.00
6	10.67	10.00	12.00	9.00	10.00	12.50	6.50	9.00	12.00	6.50
7	8.00	8.00	8.00	10.50	12.00	25.00	8.00	10.00	12.00	10.00
8	0.00	4.00	12.00	0.00	0.00	10.00	0.00	0.00	0.00	0.00
9	8.50	8.00	9.00	8.75	8.50	3.00	7.00	9.00	11.00	2.67
10	12.00	12.00	10.50	12.00	10.50	3.00	8.00	12.00	12.00	4.00
11	12.00	8.00	12.00	12.00	12.00	2.75	10.00	11.00	9.75	3.00
12	10.67	9.67	12.00	12.00	12.00	12.50	12.00	12.00	12.00	10.00
**Long-Term Survivors**										
13	7.00	8.00	6.00	8.00	6.00	9.33	8.00	8.00	8.00	2.00
14	6.00	6.00	4.50	8.00	8.50	1.00	8.00	3.50	6.00	0.00
15	8.00	12.00	8.00	12.00	10.00	10.00	12.00	12.00	6.00	10.00
16	8.00	8.00	8.50	8.00	8.00	10.00	8.00	8.00	12.00	8.00
17	7.00	6.50	6.67	12.00	8.00	6.00	7.50	11.00	8.00	5.50
18	8.00	8.00	8.00	10.00	11.00	9.00	5.75	11.00	12.00	5.50
19	4.00	7.00	7.00	8.00	10.00	4.50	7.00	11.00	9.00	6.00
20	0.00	8.00	2.00	4.00	8.00	2.00	4.00	6.00	8.00	6.00
21	5.75	7.00	8.00	11.00	7.50	2.00	7.00	10.00	7.00	6.00
22	6.67	6.33	8.00	6.33	8.67	15.00	10.67	12.00	6.00	3.67
23	1.50	5.00	7.00	6.00	10.00	5.50	7.00	10.00	7.00	8.00
24	1.50	5.33	2.00	4.00	1.50	13.50	5.00	1.00	10.00	8.00
25	5.33	9.33	6.67	9.33	8.00	3.33	8.00	9.33	8.00	3.67
26	7.00	3.50	9.75	7.25	6.25	1.50	3.50	6.75	5.25	6.00
27	5.50	9.33	5.33	8.25	10.00	8.75	8.00	10.67	6.50	2.67
28	4.00	4.00	8.67	8.00	9.33	1.33	1.33	6.67	8.67	6.00
29	10.67	8.00	5.33	10.67	8.00	26.00	4.00	8.00	12.00	5.33
30	11.00	9.00	11.00	6.00	10.00	7.75	5.00	9.00	12.00	3.75
31	4.50	6.67	4.67	7.33	7.00	30.00	4.00	6.00	10.00	7.67
32	0.00	0.00	n.d.	12.00	0.00	5.00	0.00	0.00	0.00	0.00

n.d. = not determined.

**Table 2:** Data comparison of short-term survivors vs. long-term survivors.

Antibody	Short-Term Survivors	Long-Term Survivors	*p*-Value	Cut-Off Point	Validity
Anti-CD271	8.81 (6.61–11.0; CI 95%)	5.57 (4.12–7.02; CI 95%)	0.01	8	80%
Anti-GLDC	8.76 (7.22–10.29; CI 95%)	6.85 (5.66–8.04; CI 95%)	0.05	8	73.6%
Anti-ERRFI1	10.57 (9.52–11.61; CI 95%)	6.69 (5.56–7.82; CI 95%)	<0.001	9	92.1%
Anti-TWIST	9.41 (7.30–11.52; CI 95%)	8.31 (7.17–9.44; CI 95%)	0.29	n.d.	n.d.
Anti-MSX1	8.81 (6.61–11.0; CI 95%)	7.79 (6.49–9.08; CI 95%)	0.07	n.d.	n.d.
Ki67	10.14 (5.64–14.64)	8.58 (4.92–12.22; CI 95%)	0.57	n.d.	n.d.
Anti-PTPRF	7.49 (5.13–9.86; CI 95%)	6.19 (4.83–7.55; CI 95%)	0.28	n.d.	n.d.
Anti-TNFRSF21	9.03 (6.96–11.09; CI 95%)	8 (6.4–9.6; CI 95%)	0.41	n.d.	n.d.
Anti-TNFRSF12a	9.31 (6.92–11.71; CI 95%)	8.07 (6.71–9.43; CI 95%)	0.3	n.d.	n.d.
Anti-IGFBP2	5.07 (3.01–7.13; CI 95%)	5.19 (3.95–6.43; CI 95%)	0.91	n.d.	n.d.

CI = confidence interval; n.d. = not determined.

**Table 3:** IHC scores for each antibody (high-risk group).

Patient Number	Anti-CD271	Anti-GLDC	Anti-ERRFI1	Anti-TWIST	Anti-MSX1	Anti-Ki67	Anti-PTPRF	Anti-TNFRSF21	Anti-TNFRSF12A	Anti-IGFBP2
**High-Risk Group A (Short Term until Metastasis)**										
33	7.33	7.33	8.00	7.00	9.33	6.00	6.33	10.67	9.00	9.33
34	10.00	12.00	12.00	12.00	8.00	5.00	9.00	10.00	8.00	12.00
35	10.00	12.00	8.00	12.00	12.00	2.00	8.00	10.00	12.00	12.00
36	8.00	12.00	12.00	n.d.	12.00	0.00	4.00	8.00	12.00	12.00
37	5.50	7.00	6.67	6.00	8.00	n.d.	4.00	8.00	12.00	8.00
38	10.00	10.00	8.00	10.00	8.00	1.00	10.00	10.00	12.00	8.00
39	7.00	10.00	12.00	10.00	7.00	2.00	6.00	10.00	12.00	8.00
40	12.00	9.33	9.33	9.33	6.00	13.33	9.33	9.33	8.00	10.00
41	10.00	12.00	8.00	12.00	12.00	n.d.	12.00	12.00	12.00	8.00
42	9.33	6.67	6.67	9.33	7.33	7.00	9.33	9.33	10.67	12.00
43	5.00	8.00	7.33	6.67	7.33	11.67	4.33	5.00	8.00	4.67
44	8.67	12.00	10.00	12.00	12.00	7.00	12.00	12.00	12.00	11.00
45	6.00	8.00	12.00	8.00	12.00	6.50	5.00	10.00	10.00	3.00
46	11.00	12.00	10.67	8.00	12.00	25.00	12.00	12.00	9.33	9.33
47	12.00	8.00	8.00	12.00	8.00	15.00	8.00	12.00	8.00	1.00
48	n.d.	n.d.	9.00	12.00	12.00	0.50	4.00	n.d.	n.d.	4.00
**High-Risk Group B (Long Term until Metastasis)**										
49	9.33	8.00	4.67	9.33	10.00	1.00	9.33	10.00	10.00	8.00
50	6.33	3.00	8.00	5.00	6.25	1.33	4.00	5.25	10.00	6.50
51	n.d.	8.00	n.d.	9.00	6.00	30.00	3.00	n.d.	8.00	6.00
52	n.d.	6.00	12.00	8.00	12.00	15.33	8.00	12.00	12.00	0.00
53	6.00	8.00	12.00	10.00	12.00	6.67	6.00	8.00	10.67	9.33
54	n.d.	0.00	0.00	4.00	n.d.	1.00	12.00	8.00	8.00	8.00
55	9.00	10.00	7.00	7.50	6.25	6.25	7.50	8.50	7.00	4.00

n.d. = not determined.

**Table 4:** Data comparison of high-risk group A (short term until metastasis) vs. high-risk group B (long term until metastasis).

Antibody	High-Risk Group A	High-Risk Group B	*p*-Value	Cut-Off Point	Validity
Anti-TWIST	9.76 (8.53–10.97; CI 95%)	7.55 (5.46–9.64; CI 95%)	0.04	12	75.2%
Anti-GLDC	9.76 (8.58–10.93; CI 95%)	6.14 (2.92–9.37; CI 95%)	0.007	9.33	62%
Anti-MSX1	9.56 (8.33–10.8; CI 95%)	8.75 (5.68–11.82; CI 95%)	0.5	n.d.	n.d.
Anti-CD271	8.79 (7.55–10.03; CI 95%)	7.67 (4.9–10.44; CI 95%)	0.37	n.d.	n.d.
Anti-ERRFI1	9.23 (8.18–10.27; CI 95%)	7.28 (2.47– 12.09; CI 95%)	0.35	n.d.	n.d.
Ki67	7.47	8.80	*u*-value = 1	n.d.	n.d.
Anti-PTPRF	7.71 (6.13–9.3; CI 95%)	7.12 (4.25–9.98; CI 95%)	0.67	n.d.	n.d.
Anti-TNFRSF21	9.89 (8.84–10.94; CI 95%)	8.63 (6.26–10.99; CI 95%)	0.21	n.d.	n.d.
Anti-TNFRSF12a	10.33 (9.35–11.32; CI 95%)	9.38 (7.75–11.02; CI 95%)	0.25	n.d.	n.d.
Anti-IGFBP2	13.53	8.5	*u*-value = 0.1	n.d.	n.d.

CI = confidence interval; n.d. = not determined.

**Table 5:** Primers used for gene expressions.

Symbol		Primer Sequence	Accession Number
18S	F:	GAGGATGAGGTGGAACGTGT	X03205.1
	R:	TCTTCAGTCGCTCCAGGTCT	
MSX1	F:	TCCTCAAGCTGCCAGAAGAT	P28360
	F:	TACTGCTTCTGGCGGAACTT	
TNFRSF12A	F:	CTGGCTCCAGAACAGAAAGG	Q9NP84
	R:	GGGCCTAGTGTCAAGTCTGC	
GLDC	F:	GGACCGGCCTTATTCCAGAG	P23378
	R:	TCATCAATCCGGGCAATCGT	
ERRFI	F:	TCTAGGCCTCTTCCACCGTT	Q9UJM3
ERRFI	R:	CGCCTGCCAGGAACATCATA	
IGFBP2	F:	CCTCAAGTCGGGTATGAAGG	P18065
IGFBP2	R:	ACCTGGTCCAGTTCCTGTTG	
PTPRF	F:	CAGAGGAGTCCGAGGACTATGA	P10586
PTPRF	R:	ACTGCACCTGTTGTAGTGACA	
TWIST1	F:	TCTCAAGAGGTCGTGCCAAT	Q15672
TWIST1	R:	ATGGTTTTGCAGGCCAGTTT	

**Table 6:** Clinical data of stage I6 melanoma short-term survivors and long-term survivors.

Patient Number	Sex	Age (Initial Diagnosis)	Stage at Initial Diagnosis	Breslow Tumor Thickness of Primary Melanoma (in mm)	Clark Level of Primary Melanoma	M-Classification (2009)	Month Stage I.II until Metastasis	Month Between Stage IV and Death	Localization of Analyzed Metastasis
**Short-Term Survivors**									
1	male	72	III	n.d.	n.d.	M1b	0	9	soft tissue
2	male	51	II	6	5	M1b	14	11	lung tissue
3	male	23	III	n.d.	n.d.	M1a	0	11	subcutaneous
4	female	54	I	1.7	4	M1c	37	9	bone
5	male	41	II	1.4	n.d.	M1a	13	5	skin
6	male	51	III	2.8	4	M1b	0	10	skin
7	male	43	IV	1.8	4	M1c	0	7	lymph node
8	female	69	IV	n.d.	n.d.	M1c	0	7	skin
9	male	51	III	2.7	n.d.	M1c	0	7	soft tissue
10	female	78	II	4.3	4	M1b	0	6	skin
11	female	67	III	3.5	4	M1b	0	11	lung tissue
12	male	28	I	0.7	2	M1b	116	6	skin
**Long-Term Survivors**									
13	female	47	II	n.d.	n.d.	M1a	n.d.	60	skin
14	female	70	n.d.	n.d.	4	M1a	19	61	skin
15	male	55	III	1.8	4	M1a	33	43	skin
16	male	62	I	1.9	3	M1b	9	34	lung tissue
17	female	51	II	7.0	4	n.d.	0	37	lymph node
18	female	60	III	3.2	4	M1a	0	47	skin
19	male	52	IV	n.d.	n.d.	M1a	74	49	subcutaneous
20	male	60	II	5.0	4	M1a	0	58	skin
21	female	75	II	11.7	5	M1b	105	70	lymph node
22	male	75	II	1.3	2	M1c	0	58	skin
23	female	54	II	2.0	4	M1c	0	61	lymph node
24	female	84	III	1.6	4	M1a	10	43	skin
25	male	34	II	2.5	4	M1a	0	40	skin
26	male	47	III	3.8	4	M1a	0	45	lymph node
27	male	70	I	1.4	3	n.d.	1	68	skin
28	female	68	I-II	n.d.	n.d.	M1c	3	80	lymph node
29	male	66	II	4.5	4	M1b	1	104	lymph node
30	female	66	II	5.0	4	M1a	1	61	skin
31	female	61	II	3.1	4	M1c	n.d.	31	skin
32	female	68	I	1.5	4	n.d.	n.d.	80	skin

n.d. = not determined.

**Table 7:** Clinical data of patients with high-risk primary melanoma and short term until metastasis (high-risk group A) and long term until metastasis (high-risk group B).

Patient-Number	Sex	Age at Initial Diagnosis	Stage II until First Metastasis (in Month)	Months Between First Diagnosis and Analyzed Tissue	Tumor	Localization	Breslow Tumor Thickness of Primary Melanoma (in mm)	Clark Level of Primary Melanoma	Mitotic Rate (Mitosis/mm²) of Primary Melanoma	Ulceration of Primary Melanoma	Stage at Initial Diagnosis
**High-Risk Group A (Short Term until Metastasis)**											
33	male	71	6	0	primary melanoma	mucosa	5	n.d.	n.d.	yes	II
34	female	76	1	0	primary melanoma	skin	9.2	n.d.	n.d.	yes	II
35	male	61	0	0	primary melanoma	skin	6.8	4	n.d.	yes	III
36	female	64	0	0	primary melanoma	skin	3.5	n.d.	n.d.	yes	II
37	male	40	0	0	primary melanoma	skin	3.6	3	6	yes	III
38	male	53	0	0	primary melanoma	skin	3.5	3	n.d.	no	III
39	male	75	5	0	primary melanoma	skin	2.8	4	8	no	II
40	male	90	6	0	primary melanoma	skin	7.7	5	9	yes	II
41	female	81	1	0	primary melanoma	skin	3.5	4	n.d.	yes	II
42	male	65	5	0	primary melanoma	skin	6.5	4	10	yes	II
43	male	41	0	0	primary melanoma	skin	3.2	4	n.d.	n.d.	III
44	male	88	0	0	primary melanoma	skin	5	4	n.d.	n.d.	III
45	male	51	0	0	primary melanoma	skin	2.8	4	n.d.	n.d.	III
46	male	77	5	0	primary melanoma	skin	9	5	n.d.	n.d.	II
47	male	66	3	0	primary melanoma	skin	4.5	4	n.d.	n.d.	II
48	female	67	0	0	primary melanoma	skin	3.5	4	n.d.	n.d.	III
**High-Risk Group B (Long Term until Metastasis)**											
49	male	65	40	0	primary melanoma	skin	2.1	n.d.	2	n.d.	II
50	male	78	31	0	primary melanoma	skin	2.5	n.d.	n.d.	yes	II
51	male	75	47	0	primary melanoma	skin	2.6	4	n.d.	no	II
52	female	66	34	0	primary melanoma	skin	2.4	4	n.d.	yes	II
53	male	48	39	0	primary melanoma	skin	2.2	4	7	no	II
54	female	76	32	0	primary melanoma	skin	3.3	4	n.d.	no	II
55	male	70	39	0	primary melanoma	skin	2.3	4	n.d.	yes	II

n.d. = not determined.

## Supplementary Materials

The following are available online at http://www.mdpi.com/2072-6694/11/1/76/s1, Figure S1: IHC score of short-term survival and long-term survival. IHC score analyses of the two subgroups (short-term survival and long-term survival) are displayed in this chart. Y-axis shows the overall IHC score (0–12), which was calculated as the product of quantity and intensity of the staining. Displayed antibodies did not show significant difference. Figure S2: IHC score in high-risk group A and high-risk group B. IHC score analyses of the two subgroups (high-risk group A and high-risk group B) are displayed in this chart. Y-axis shows the overall IHC score (0–12), which was calculated as the product of quantity and intensity of the staining (not significant). Figure S3: Intensity of TMA stainings. Immunohistochemical stainings are shown for survival group (CD271, GLDC, and ERRFI1) in S100-β-positive melanoma cells. Samples with, mild, moderate, and strong intensity (12× and 80× magnifications) are shown. Figure S4: Intensity of TMA stainings. Immunohistochemical stainings with mild, moderate, and strong intensity are shown for Anti-MSX1, Anti-TWIST, Anti-TNFRSF12a, Anti-IGFBP2, Anti-TNFRSFR21, and Anti-PTPRF in S100-β-positive sections (12× and 80× magnifications are shown).
