# Supplementary material for: Expression of Neural Crest Markers GLDC and ERRFI1 is Correlated with Melanoma Prognosis
Source: Cancers (Basel). 2019 Jan 11;11(1):76. doi: 10.3390/cancers11010076 (PMC6356846; doi:10.3390/cancers11010076)
Supplement: Supplementary file 1 [file cancers-11-00076-s001.pdf]

Supplementary Materials: Expression of Neural Crest Markers GLDC and ERRFI1 is Correlated with Melanoma Prognosis

Katharina Jäger, Lionel Larribère, Huizi Wu, Christel Weiss, Christoffer Gebhardt and Jochen Utikal

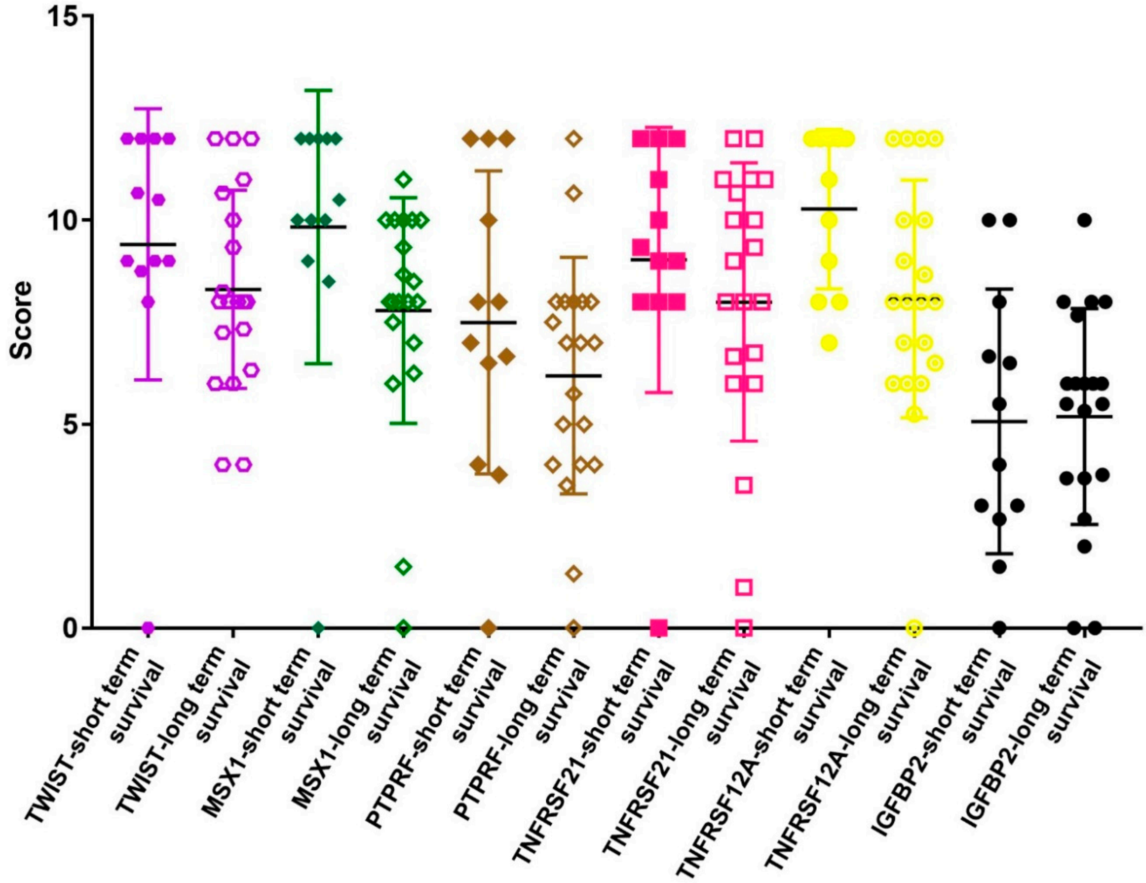

Figure S1. IHC score of short-term survival and long-term survival. IHC score analyses of the two subgroups (short-term survival and long-term survival) are displayed in this chart. Y-axis shows the overall IHC score (0–12), which was calculated as the product of quantity and intensity of the staining. Displayed antibodies did not show significant difference.

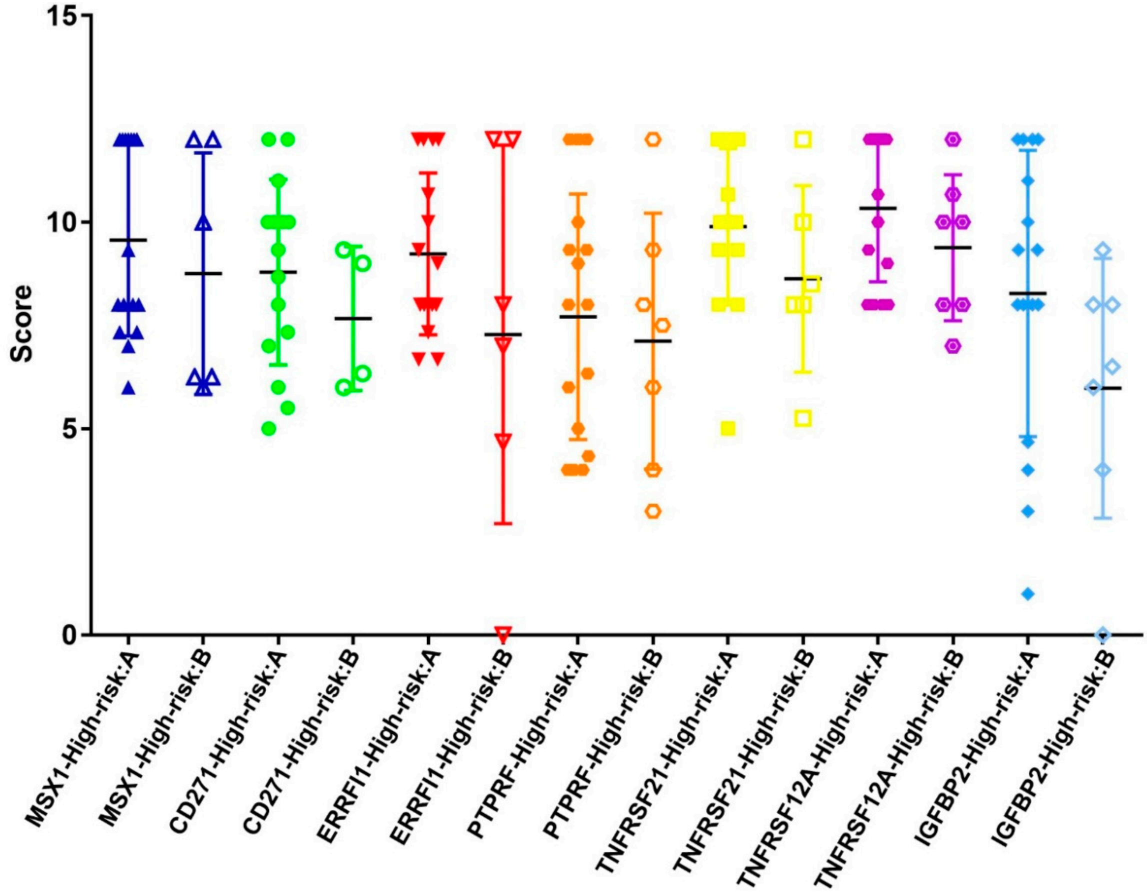

Figure S**2.** IHC score in high-risk group A and high-risk group B. IHC score analyses of the two subgroups (high-risk group A and high-risk group B) are displayed in this chart. Y-axis shows the overall IHC score (0–12), which was calculated as the product of quantity and intensity of the staining (not significant).

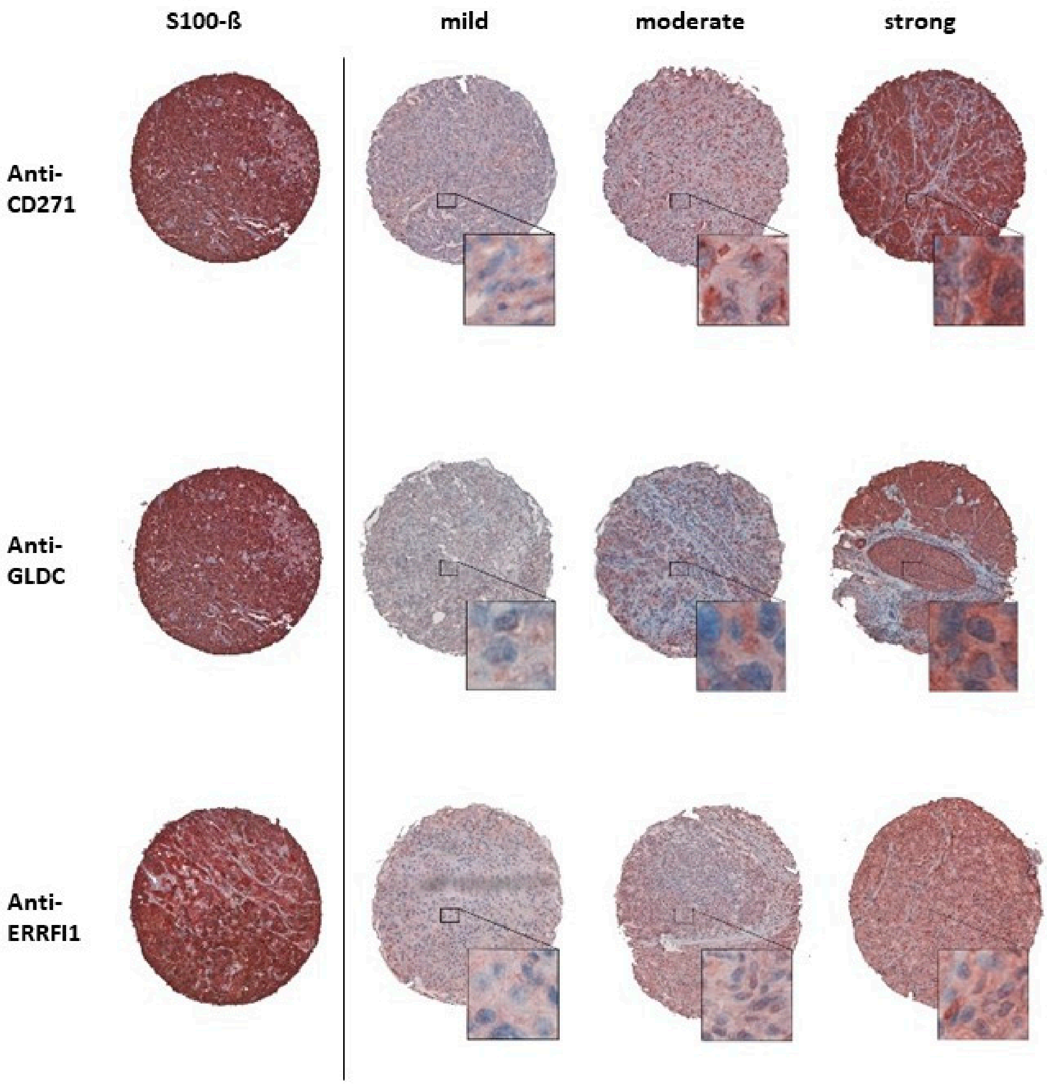

Figure S3. Intensity of TMA stainings. Immunohistochemical stainings are shown for survival group (CD271, GLDC, and ERRFI1) in S100-ß-positive melanoma cells. Samples with, mild, moderate, and strong intensity (12× and 80× magnifications) are shown.

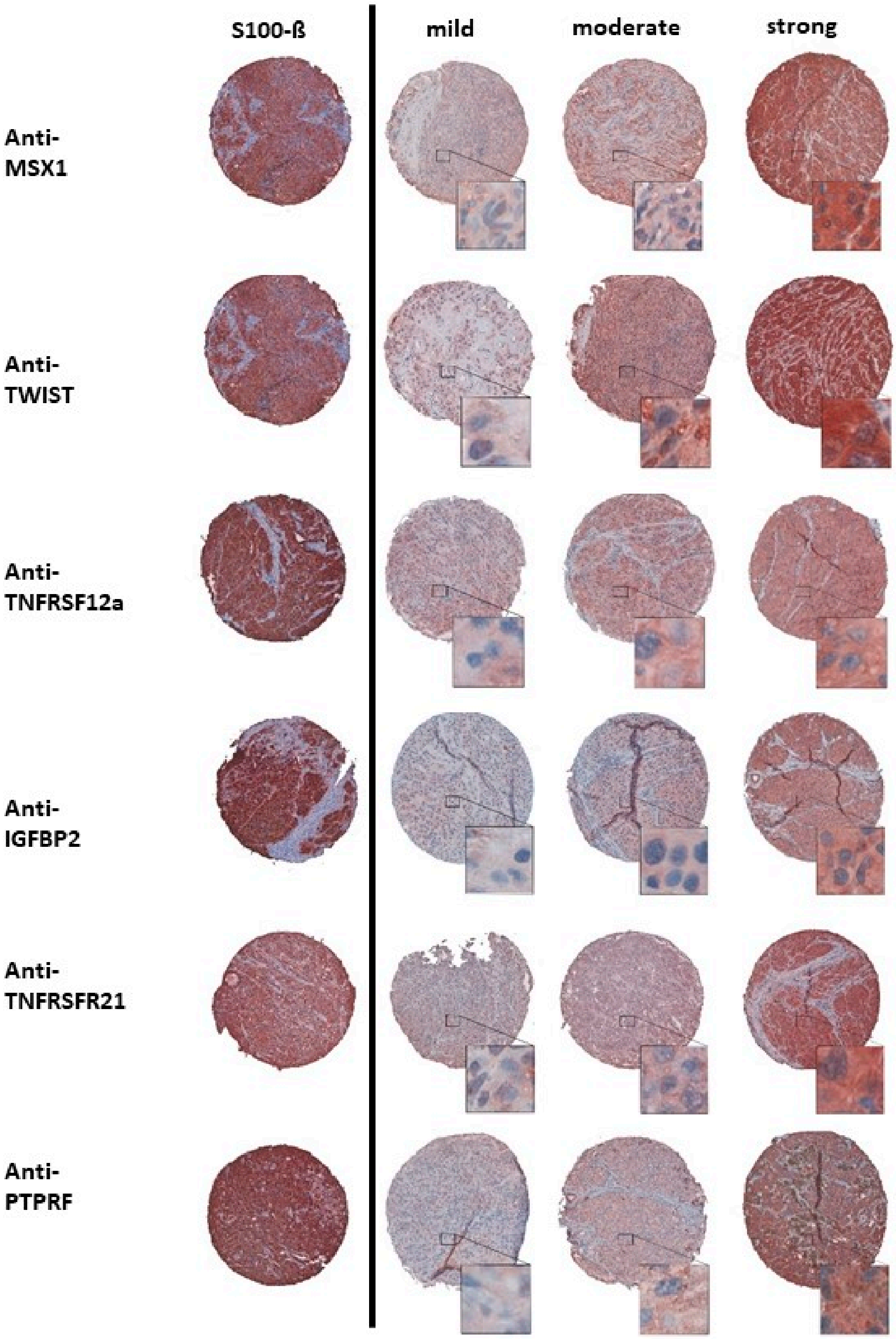

Figure S4. Intensity of TMA stainings. Immunohistochemical stainings with mild, moderate, and strong intensity are shown for Anti-MSX1, Anti-TWIST, Anti-TNFRSF12a, Anti-IGFBP2, Anti-TNFRSFR21, and Anti-PTPRF in S100-ß-positive sections (12× and 80× magnifications are shown).

© 2018 by the authors. Licensee MDPI, Basel, Switzerland. This article is an open access article distributed under the terms and conditions of the Creative Commons Attribution (CC BY) license (http://creativecommons.org/licenses/by/4.0/).
